# Optimal model-based sensorless adaptive optics for epifluorescence microscopy

**DOI:** 10.1371/journal.pone.0194523

**Published:** 2018-03-20

**Authors:** Paolo Pozzi, Oleg Soloviev, Dean Wilding, Gleb Vdovin, Michel Verhaegen

**Affiliations:** 1 Delft Center for Systems and Control, Delft University of Technology, Delft, The Netherlands; 2 Flexible Optical B.V., Rijswijk, The Netherlands; 3 ITMO University, St Petersburg, Russian Federation; University of California San Francisco, UNITED STATES

## Abstract

We report on a universal sample-independent sensorless adaptive optics method, based on modal optimization of the second moment of the fluorescence emission from a point-like excitation. Our method employs a sample-independent precalibration, performed only once for the particular system, to establish the direct relation between the image quality and the aberration. The method is potentially applicable to any form of microscopy with epifluorescence detection, including the practically important case of incoherent fluorescence emission from a three dimensional object, through minor hardware modifications. We have applied the technique successfully to a widefield epifluorescence microscope and to a multiaperture confocal microscope.

## Introduction

Adaptive optics can be employed in fluorescence microscopy to compensate for system or sample induced phase aberrations in the optical path, increasing the quality of the acquired images, especially in thick and turbid samples. Due to the low brightness of fluorescence emission and to the absence of guide stars in most samples, the correction of aberrations is generally performed through the optimization of an image-based metric. A common method for metric optimization is a model-based procedure, using a-priori assumptions on the dependence of the metric from the aberration, providing fast convergence speed with good reliability. Model-based aberration correction in fluorescence microscopy techniques is usually performed [1–3] with a hill climbing optimization procedure, fitting of an image-based performance metric *M* within an *N*-dimensional aberration space generated by a base

$$X=\{x_{1},x_{2},\ldots ,x_{N}\}$$ (1)

of aberration functions.

For any base *X*, any phase aberration, neglecting its piston component, can be described as

$$\varphi =\sum_{n=1}^{N}a_{n}x_{n}.$$ (2)

The value of a well chosen metric can be approximated, for small aberrations, as a quadratic model:

$$M\left(\varphi \right)\approx M_{0}-a^{T}Ba,$$ (3)

where **B** ≽ 0 is a constant, positive semi-definite *N* × *N* matrix, and *a* is the vector of coefficients, *a*^*T*^ = (*a*_1_, …, *a*_*n*_).

In the particular case in which matrix B is diagonal, the metric function can be expressed as

$$\begin{array}{c} M\left(\varphi \right)=M_{0}-\sum_{n=1}^{N}b_{n}a_{n}^{2}, \end{array}$$ (4)

and correction of the wavefront through an adaptive optical element (AOE), such as a deformable mirror (DM), can be performed on each element of the base separately through a quadratic fit, therefore requiring only 2*N* + 1 measurements of *M* for full correction of any aberration.

The main drawback in the use of such a correction method is that, given an image metric *M*, a generic base for aberration space, such as the Zernike polynomials or the influence functions of the actuators of an AOE, generally does not satisfy condition (4). Moreover, the quadratic model of function (3), depending on the metric used, is generally only valid for small aberrations [4].

Several methods have been devised to generate, given a metric *M*, a suitable base *X* satisfying condition (4). In general the methods are based on a practical calibration procedure involving *O*(*N*^2^) measurements of the metric *M* on a sample [5]. This may only work for small aberrations, and is based on the reasonable but generally not rigorously proven assumption that after calibration on a given field of view, condition (4) will be satisfied by the same base and metric function in any other field of view in the sample.

In this paper we propose a new approach to model-based hill-climbing optimization in fluorescence microscopy, involving small hardware modifications of the microscope, in order to enable measuring of a sample independent metric. The modification applied allows to measure the second moment of the fluorescence emission distribution from a point-like excitation spot, which constitutes a sample independent metric. Furthermore, we prove that such a metric respects condition (4) for an analytically defined base set (gradient-orthogonal aberrations), for aberrations of any amplitude. Finally, we present a method for calibrating any AOE with a reference source and a wavefront sensor, in order to generate such modes without requiring sample dependent calibration procedures. The main practical advantages of the method are its validity for aberrations of any amplitude, and the speed and reliability of the wavefront sensor-based calibration procedure, as compared to image-based calibrations, which are generally slower, and can be severely affected by noise.

Second moment methods [6] have already been proven ideal for hill-climbing optimization in regular sensorless adaptive optics, when imaging the point spread function of the system from a coherent point-like source [7]; However, in order to successfully prove the validity of this method for the case of fluorescence microscopy in thick samples, the model should be extended to incoherent emission from a three dimensional source, (see section “Physical model and proof of validity”).

Alternatively, image-based metrics, in particular the total intensity of a selected frequency range of the power spectrum of the image, have been shown to respect condition 4 for gradient-orthogonal bases, such as the Lukosz polynomials [8, 9]. The main advantage in using image-based methods, compared to the method proposed in this paper, is the relatively simpler hardware setup, where the only modification required to the optical setup is the addition of the AOE. However, such methods work only for small aberrations, as the dependence of the metric from the aberration is Lorentzian, and can only be approximated with a quadratic function in a small range. Additionally, the frequency range considered must be carefully calibrated based on the nature of the sample, and especially on the contribution of out of focus fluorescence emission. Moreover, they would not work for aperture-based scanning setups, as the physical image formation process is different.

Due to these limitations, to the knowledge of the authors, calibration-less image-based methods have never been reported working on thick fluorescent samples, but only on brightfield images of bidimensional samples [8], or on coherent images of pure scatterers [9].

## Correction technique

The correction technique can be applied in any fluorescence microscope based on camera detection (e.g. Epifluorescence microscopy, Structured illumination microscopy, lightsheet microscopy, localization superresolution microscopy), with the addition of a secondary excitation source generating an array of point-like spots in the image plane. This can be achieved with a wide variety of methods such as the use of an incoherent source and a digital micromirror device or pinhole array, or with a coherent source and a diffractive optical element or microlens array.

**Fig 1:**
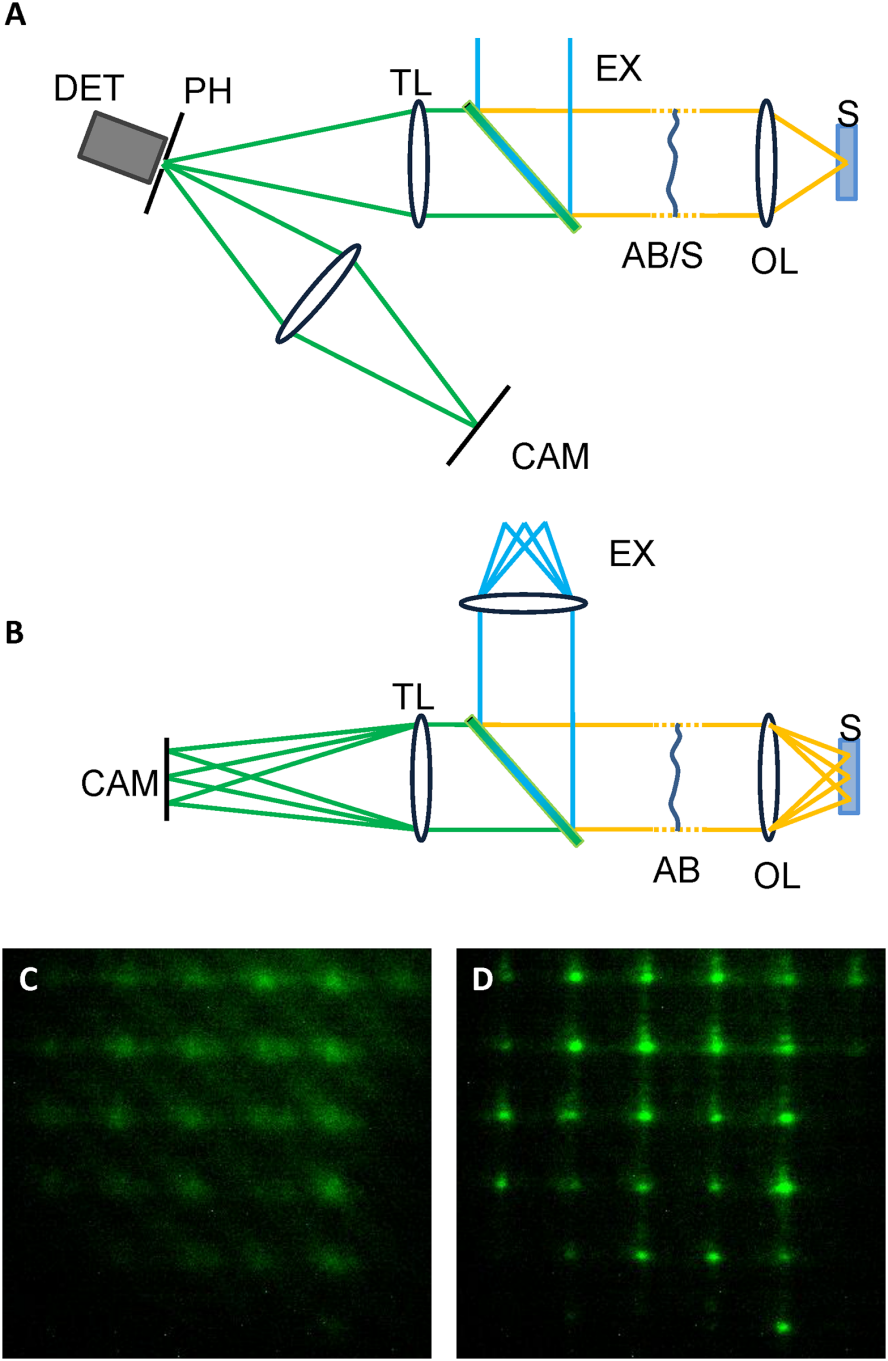
Simplified schematic of the proposed experimental setups. A: Pinhole based confocal microscopes, and B: camera based microscopes. EX—Excitation source, AB/S—Aberration correction (and scanning), OL—Objective lens, S—Sample, TL—Tube lens, PH—Pinhole, DET—detector, CAM—camera. C, D: Example image of fluorescence spots in the presence of an aberration in C, and after correction in D. The metric employed in the optimization is the second moment of the average image of the spots.

In alternative, the technique can be applied to a scanning confocal fluorescence microscope, with single (e.g. confocal laser scanning microscopy, STED microscopy) or multiple (e.g. Spinning disk microscopy, programmable array microscopy) apertures, by using a confocal aperture created on a reflective surface. The aperture surface should be slightly tilted with respect to the optical axis, in order to allow for the reflection of fluorescence light to be imaged on a camera, as shown in Fig 1.

In order to correct aberrations, an AOE should be present in a pupil plane of the system shared by excitation and emission light. The AOE should be pre-calibrated with a wavefront sensor, in order to introduce aberrations described by coefficients of a gradient orthogonal base [8] (rigorously defined in the section “Physical model and proof of validity”).

The metric used is the second moment of the spatial distribution at the image plane of fluorescence emission from a diffraction limited, point-like excitation source, averaged over multiple positions in the field of view.

Each measurement of the metric should be performed on the average of images of point-like excitation spots in several locations in the field of view. This would happen differently in a camera based system or in a aperture based confocal microscope.

In a single aperture microscope, shown in Fig 1A, the pinhole can be exposed for the entire duration of the image scan. Then the metric can be computed by averaging the pinhole images, sampled in arbitrary number of bright pixels evenly distributed in the field of view, in a “random access” fashion [10]. In a multi-aperture confocal microscope, a similar procedure can be applied using the rejected light image.

In a camera based system, shown in Fig 1B, once a pattern is projected, the brightest spots should be selected and averaged on a single image.

Correction of an aberration can then be performed as described in the introduction, by acquiring for each element *x*_*n*_ of the gradient orthogonal base the three measurements of the second moment of the distribution *M*_0_ = *M*(*φ*), *M*_+_ = *M*(*φ* + *ax*_*n*_) and *M*_−_ = *M*(*φ* − *ax*_*n*_), where *a* is an arbitrarily chosen factor, and determining the optimal correction as the minimum of the quadratic fit of the three points. Since the measurement of *M*_0_ is the same for all elements of the base, the procedure only requires 2*N* + 1 measurements.

## Physical model and proof of validity of the method

**Definition of image moments**. Describing an image as a bidimensional distribution of light intensity *I*(*x*, *y*) in a field of view *F*, its first moment, or center of mass, is defined as

$$\begin{array}{c} \left\{m_{1x}\left(I\right),m_{1y}\left(I\right)\right\}=\{\frac{\int_{F}I\left(x,y\right)\cdot x\;dx\;dy}{\int_{F}I\left(x,y\right)\;dx\;dy},\frac{\int_{F}I\left(x,y\right)\cdot y\;dx\;dy}{\int_{F}^{}I\left(x,y\right)\;dx\;dy}\}, \end{array}$$ (5)

and the central second moment sm is defined as

$$\begin{array}{c} \text{sm}\left(I\right)=\frac{\int_{F}I\left(x,y\right)({\left(x-m_{1x}\left(I\right)\right)}^{2}+{\left(y-m_{1y}\left(I\right)\right)}^{2})\;dx\;dy}{\int_{F}I\left(x,y\right)\;dx\;dy}. \end{array}$$ (6)

**Definition of gradient orthogonal base.** Given two phase aberrations *φ*_1_ and *φ*_2_, a gradient dot product operator ∴ can be defined as the dot product between the gradients of *φ*_1_ and *φ*_2_

$$\begin{array}{c} \varphi_{1}∴\varphi_{2}=\int_{P}^{}\nabla \varphi_{1}\cdot \nabla \varphi_{2}\;dx\;dy. \end{array}$$ (7)

A gradient-orthogonal base *G* = {*g*_1_, *g*_2_, …} is a base in which, for any couple of vectors of the base *g*_*i*_ ∴ *g*_*j*_ ∝ *δ*_*ij*_, where *δ* is the Kronecker delta. It is important to notice that

$$\begin{array}{c} \underset{P}{\int }{\left|\nabla \left(g_{i}+g_{j}\right)\right|}^{2}\text{d}x\;\text{d}y=\underset{P}{\int }{\left|\nabla \left(g_{i}\right)\right|}^{2}\text{d}x\;\text{d}y+\underset{P}{\int }{\left|\nabla \left(g_{j}\right)\right|}^{2}\text{d}x\;\text{d}y,\;\text{for}\;i\neq j. \end{array}$$ (8)

A practical consideration is that the displacement Zernike polynomials *Z*_2_, *Z*_3_, and *Z*_4_ (tip, tilt, and defocus) verify the previous condition. A convenient gradient-orthogonal base should therefore include these three displacement modes, and aberration correction should be performed on all remaining modes, to ensure that the correction procedure does not displace the field of view.

**Fig 2:**
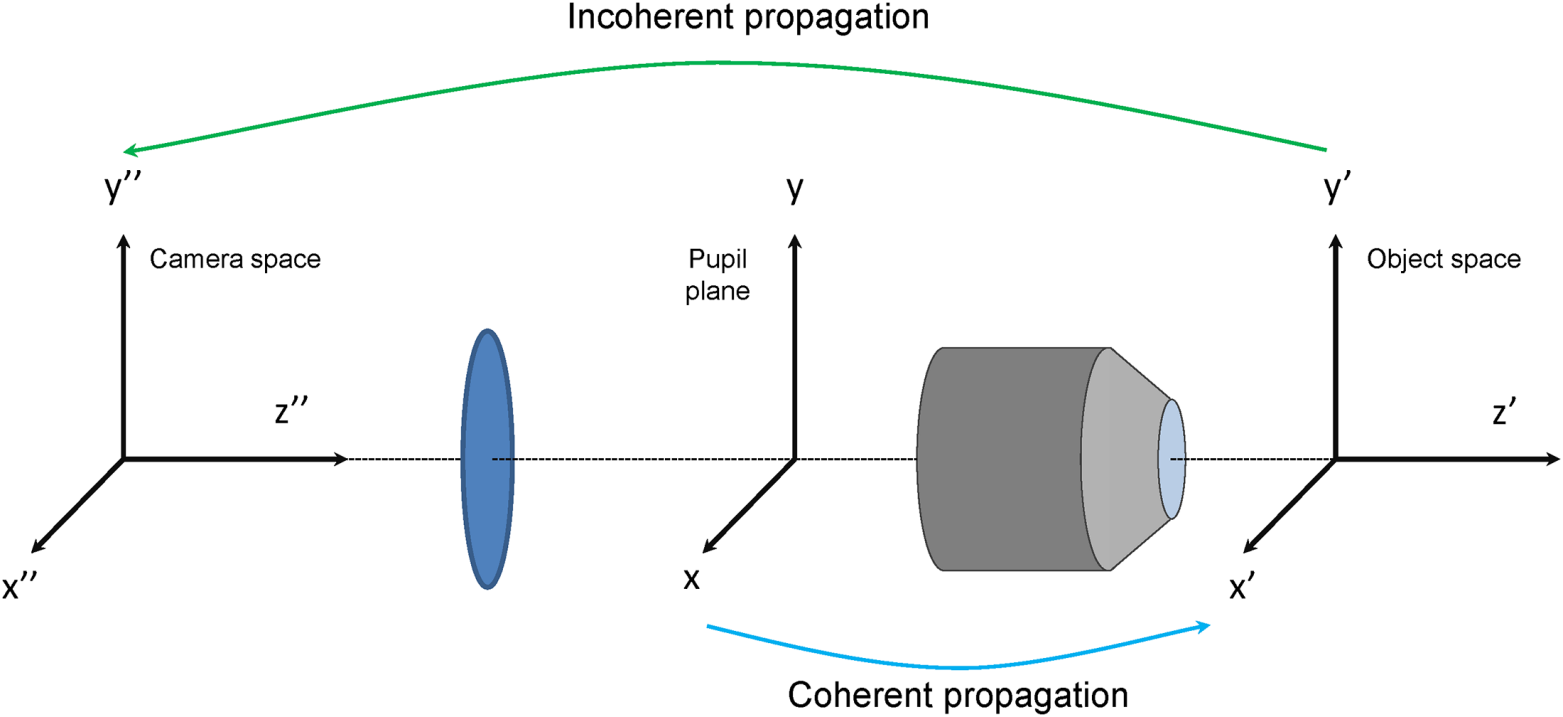
Scheme of the physical model of a microscope. A coherent illumination with an aberration is considered in the pupil plane of the system, and coherently propagated to the object space to obtain the illumination point spread function. Fluorescence emission is calculated as multiplication of the excitation point spread function with the object distribution. Incoherent propagation to camera space is calculated as the convolution of fluorescence emission with the illumination point spread function.

**Physical model of the optical system.** A generic microscopy setup is modeled as a telescope system formed by a tube lens and an objective lens. A scheme reporting the conventions in the systems of coordinates used is reported in Fig 2. The fluorescence image *I*_cam_(*x*″, *y*″) of a point-like excitation spot is dependent on the phase aberration *φ*(*x*, *y*) at the pupil plane, on the aperture of the optical system, and on the three-dimensional distribution *O*(*x*′, *y*′, *z*′) of fluorophore concentration at the sample.

In particular, consider a field at the pupil plane

$$E_{p}=P\left(x,y\right)e^{i\varphi },$$ (9)

where *P*(*x*, *y*), in the assumption of evenly illuminated pupil, is the aperture function

$$\begin{array}{c} P\left(x,y\right)=\{\begin{array}{c} 1,\;x^{2}+y^{2}\leq D^{2}\\ 0,\;x^{2}+y^{2}>D^{2} \end{array}. \end{array}$$ (10)

The field *E*_o_(*x*′, *y*′, *z*′) of excitation light at the object coordinates is approximated by coherent propagation as

$$\begin{array}{c} E_{o}\left(x^{\prime },y^{\prime },z^{\prime }\right)\approx e^{i\pi \frac{{x^{\prime }}^{2}+{y^{\prime }}^{2}}{\lambda f}(\frac{z^{\prime }}{f})}\int_{R^{2}}^{}E_{p}\left(x,y\right)e^{-i2\pi \frac{xx^{\prime }+yy^{\prime }}{\lambda f}}\;dx\;dy, \end{array}$$ (11)

with *f* being the focal length of the objective lens. *E*_o_, representing the complex point spread function of the system, is equal to the Fourier transform of *E*_*p*_ at the focal plane (where *z*′ = 0), and the Fourier transform of $E_{pz}=P\left(x,y\right)e^{i\varphi_{z}}$ elsewhere, where

$$\begin{array}{c} \varphi_{z}^{\prime }=\varphi +\pi \frac{x^{2}+y^{2}}{\lambda f}(\frac{z^{\prime }}{f})=\varphi +a\left(z^{\prime }\right)Z_{4}\left(x,y\right), \end{array}$$ (12)

with *Z*_4_ equal to the defocus Zernike polynomial. The distribution *I*_o_(*x*′, *y*′, *z*′) of fluorescence emission is proportional to the intensity of excitation light multiplied by the spatial distribution of the fluorophore *O*:

$$I_{\text{o}}\propto {\left|{\text{E}}_{\text{o}}\right|}^{2}\;O.$$ (13)

Due to the incoherent nature of fluorescence emission, the spatial fluorescence intensity distribution *I*_*em*_(*x*″, *y*″, *z*″) at the camera will simply be the convolution of *I*_o_ and the three dimensional point spread function of the system (the coordinates system is considered the same, neglecting the magnification of the optical system). As the aberration affecting the point spread function is still *φ*, but applied to light propagating in the opposite direction, the point spread function is equal to |*E*_o_|^2^, with inverted coordinates, leading to

$$\begin{array}{c} I_{em}\left(x^{\prime \prime },y^{\prime \prime },z^{\prime \prime }\right)=I_{\text{o}}\left(x^{\prime },y^{\prime },z^{\prime }\right)\times {\left|E_{\text{o}}\left(-x^{\prime },-y^{\prime },-z^{\prime }\right)\right|}^{2}. \end{array}$$ (14)

Since image detectors are two dimensional, and positioned at the focal plane of the system, the detected intensity *I*_*cam*_ at the camera plane can be just expressed as:

$$\begin{array}{c} I_{\text{cam}}(x^{\prime \prime },y^{\prime \prime })=I_{em}(x^{\prime \prime },y^{\prime \prime },0). \end{array}$$ (15)

It is important to notice that, considering two different samples *O*_1_(*x*′, *y*′, *z*′) and *O*_2_(*x*′, *y*′, *z*′), due to the incoherent nature of fluorescence, the sum of the corresponding images is exactly equal to the image generated by a sum of the two objects *O*_1_ + *O*_2_.

As a direct consequence, averaging on a wide enough variety of random objects, which can be achieved by averaging images in multiple excitation spots over the same field of view, is practically equivalent to sampling a single object with uniform fluorophore distribution. In this scenario, through the rest of this formal proof, we will assume illumination is generated in a single point, and the fluorophore distribution is assumed constant:

$$\begin{array}{c} O(x^{\prime },y^{\prime },z^{\prime })=1. \end{array}$$ (16)

**Proof of validity of the method**. This section of the paper demonstrates that, considering the image of fluorescence light emitted by a point-like excitation spot, the variation in second moment with respect to a diffraction limited condition can be used as a metric *M*, for which condition (4) is verified by a gradient-orthogonal base *G*.

In a previous publication [6], the authors proved that, in the presence of a phase aberration, the variation of the second moment of both the image of a coherent point source and of an incoherent two-dimensional extended source is proportional to the mean square gradient magnitude of the phase aberration. In mathematical terms, for any two-dimensional object *O*_2*D*_(*x*′, *y*′), in the presence of a phase aberration *φ*(*x*, *y*) this can be written as:

$$\begin{array}{c} \text{sm}\left(O_{2D},\varphi \right)-\text{sm}\left(O_{2D},0\right)\propto \underset{P}{\int }{\left|\nabla \varphi \right|}^{2}\text{d}x\;\text{d}y, \end{array}$$ (17)

where sm(*O*_2*D*_, *φ*) is the second moment of the intensity distribution of the image of *O*_2*D*_ when affected by the aberration *φ*, and sm(*O*_2*D*_, 0) is the second moment of the diffraction-limited image of *O*_2*D*_.

Let us now apply Eq (17) to the model described in the paragraph “Physical model of the optical system”. Defining sm_o_(*φ*, *z*′) as the second moment of excitation intensity distribution at the object location for a given aberration *φ* at an axial distance *z*′ from the image plane, Eq (17) can be written as:

$$\begin{array}{c} {\text{sm}}_{\text{o}}\left(\varphi ,z^{\prime }\right)-{\text{sm}}_{\text{o}}\left(0,0\right)=c_{\text{o}}\left(z^{\prime }\right)\underset{P}{\int }{\left|\nabla \varphi_{z^{\prime }}\right|}^{2}\text{d}x\;\text{d}y, \end{array}$$ (18)

where *φ*_*z*′_ is as defined in Eq (12), *c*_o_(*z*′) is a constant only depending on *z*′, and sm_o_(0, 0) is the second moment of excitation intensity distribution at the focal plane in the absence of aberrations.

Under assumption (16), the fluorescence emission is imaged at the camera plane as an incoherent extended source with the same intensity distribution as the excitation light.

If fluorescence emission would not be affected by any aberration, the image obtained at the camera plane would be equivalent to the extended source, and therefore the second moment sm_cam_ of intensity at the camera plane would be sm_cam_(0, *z*′) = sm_o_(*φ*, *z*′).

In the case of epifluorescence imaging, however, fluorescence light from a plane at a distance *z* from the focal plane in object space is affected by an aberration *ϑ*_*z*′_(*x*, *y*) = *φ*_*z*′_(−*x*, −*y*). Observing that

$$\underset{P}{\int }\begin{array}{c} {\left|\nabla \vartheta_{z^{\prime }}\right|}^{2}\text{d}x\;\text{d}y=\underset{P}{\int }{\left|\nabla \varphi_{z^{\prime }}\right|}^{2}\text{d}x\;\text{d}y, \end{array}$$ (19)

we can apply Eq (17) again, so that the second moment of the camera image sm_cam_(*ϑ*, *z*′) follows the rule:

$$\begin{array}{c} {\text{sm}}_{\text{cam}}(\vartheta ,z^{\prime })-{\text{sm}}_{\text{cam}}(0,z^{\prime })={\text{sm}}_{\text{cam}}(\vartheta ,z^{\prime })-{\text{sm}}_{o}(\varphi ,z^{\prime })=c_{\text{cam}}\left(z^{\prime }\right)\int_{P}^{}{\left|\nabla \varphi_{z^{\prime }}\right|}^{2}\;dx\;dy, \end{array}$$ (20)

considering Eq (18), it easily follows that

$$\begin{array}{c} {\text{sm}}_{\text{cam}}(\vartheta ,z^{\prime })-{\text{sm}}_{o}(0,0)=c\left(z^{\prime }\right)\int_{P}^{}{\left|\nabla \varphi_{z^{\prime }}\right|}^{2}\;dx\;dy, \end{array}$$ (21)

where *c*(*z*′) = *c*_cam_(*z*′) + *c*_o_(*z*′). The actual image at the camera plane is the integral over the *z*′ range of the images of the single planes. Neglecting the loss in excitation light due to absorption, and therefore considering equal total excitation power for all values of *z*′, the second moment sm of the final image is the average second moment over *z*′:

$$\begin{array}{c} \text{sm}\left(\varphi \right)=\frac{\int_{-T}^{T}c\left(z^{\prime }\right)(\int_{P}^{}{\left|\nabla \varphi_{z^{\prime }}\right|}^{2}\;dx\;dy)\;dz^{\prime }}{2T}, \end{array}$$ (22)

where *T* is defined as the distance for which

$$\begin{array}{c} {\text{sm}}_{o}(0,T)\gg {\text{sm}}_{o}(0,0). \end{array}$$ (23)

The choice of limiting the integral to *T* is due to the observation that objects too far from the depth of field of the objective will have no distinguishable details, and negligible intensity compared to the objects close to the focal plane, and can therefore be excluded from the integral. Decomposing the aberration *φ* on a gradient orthogonal base G which includes the Zernike displacement modes (in Noll’s index notation)

$$\begin{array}{c} G=\{Z_{2},Z_{3},Z_{4},g_{1},g_{2},\ldots \}, \end{array}$$ (24)

and assuming it free of displacement modes, the aberrations *φ*_*z*′_ can be described as

$$\begin{array}{c} \varphi_{z^{\prime }}=a\left(z^{\prime }\right)Z_{4}+\varphi =a\left(z^{\prime }\right)Z_{4}+\sum_{i}b_{i}g_{i}. \end{array}$$ (25)

It can therefore be derived that

$$\begin{array}{c} \text{sm}\left(\varphi \right)=\frac{\int_{-T}^{T}c\left(z^{\prime }\right)(\int_{P}^{}a^{2}\left(z^{\prime }\right){\left|\nabla Z_{4}\right|}^{2}\;dx\;dy)\;dz^{\prime }}{2T}+\frac{\int_{-T}^{T}c\left(z^{\prime }\right)dz^{\prime }\sum_{i}{b_{i}}^{2}(\int_{P}^{}{\left|\nabla g_{i}\right|}^{2}\;dx\;dy)}{2T}, \end{array}$$ (26)

Denoting the sample dependent second moment of the image in the absence of an aberration as a constant

$$\begin{array}{c} \text{sm}\left(0\right)=\frac{\int_{-T}^{T}c\left(z^{\prime }\right)\left(\int_{P}a^{2}\left(z^{\prime }\right){\left|\nabla Z_{4}\right|}^{2}\text{d}x\;\text{d}y\right)\text{d}z^{\prime }}{2T}, \end{array}$$ (27)

it can be seen that the metric *M*(*φ*) = sm(*φ*) respects condition (4) independently of the sample shape.

**Assumption and limitations**. A few assumptions were made in the physical modeling of the system, and proof of validity of the method. This section lists the most apparent, and the effect they can have on the experimental implementation of the system.

- Aberrations are assumed to be isoplanatic, meaning that a single phase aberration profile is present in the pupil plane of the system, resulting in a constant effect over the full field of view. While, considering the geometry of a sample induced aberration in microscopy system this is clearly not true, this is a common assumption in adaptive optics for microscopy. Image metrics calculated over the whole field of view are generally used, in the assumption the optimization of the metric will lead to the correction of the average aberration over the field of view. Since the metric described here is computed over multiple positions in the field of view, this assumption holds validity.
- Aberrations are considered constant over the whole thickness interval T. While it can be assumed that the order and amplitude of aberrations increases with depth in the sample, data reported in literature [11] suggests that the variation is small over the Rayleigh length of high numerical aperture objectives.
- The method is supposedly working for samples of any thickness. This is true as long as the intensity contribution to the spot image of the signal from within the distance *T* of the system is much brighter than that of out of focus light. This is true for a single, unaberrated spot, and it holds true in the presence of aberrations which can be reasonably compensated with a low order commercial AOE. This assumption could be an issue in the case of parallelized spots creation, as the contribution of multiple beams could sum up out of focus. As in any parallelized confocal system, the relationship between sample thickness, numerical aperture, and spots spacing must be carefully considered in order to successfully correct aberrations, as well as maintaining optical sectioning.
- The method proposed is mathematically valid for aberrations of any amplitude. However there are, obviously, physical limits to validity of the system. The main limit is the size of the detection area for the second moment metric, limited by the sensor size, and by the spacing between points in the case of a microscopy with multiple illumination points. High amplitude aberrations would require bigger sensors or lower magnification, and if necessary an increase in the spacing between illumination points. In an extreme situation, if the light is spread over an area too wide, the detection signal to noise ratio could be insufficient to correctly determine the second moment of the distribution.
- The method proposed, as in most adaptive optics methods for non monochromatic systems, does not consider dispersion in the sample, assuming the same aberration is present for all wavelengths in both excitation and emission spectrum. This is generally a valid assumption for most common fluorescent specimens, exhibiting small Stokes shifts, but could be a challenge for future attempts to implement the method in multiphoton excitation microscopes.

## AOE calibration procedure

In a practical setup, the gradient dot product (Eq 7) between the phase correction introduced by an AOE can be estimated from the centroids displacements on a Shack-Hartmann wavefront sensor. The wavefront sensor is only required for calibration of the mirror, which could be performed on a separate optical setup, without the need of incorporating it in the microscope. In practice, the AOE can be calibrated on a separate optical setup, and then included in the microscope. In particular, given a Shack-Hartmann sensor with *N*_sh_ centroids, each with a displacement measured in two components *dx*_*i*_ and *dy*_*i*_, introducing two aberration *φ*_1_ and *φ*_2_, the dot product can be estimated as:

$$\begin{array}{c} \varphi_{1}∴\varphi_{2}\approx \sum_{i=1}^{N_{\text{sh}}}dx_{i}(\varphi_{1})dx_{i}(\varphi_{2})+dy_{i}(\varphi_{1})dy_{i}(\varphi_{2}). \end{array}$$ (28)

In order to obtain a gradient orthogonal base expressed as a set of inputs for the *N*_act_ actuators of the AOE, the centroids displacements for each influence function *φ*_*i*_ of each actuator should be measured, and a gradient products matrix *G* of size *N*_act_ × *N*_act_ can be estimated, with each element equal to:

$$\begin{array}{c} G_{ij}=\varphi_{i}∴\varphi_{j}, \end{array}$$ (29)

estimated as in (28).

**Fig 3:**
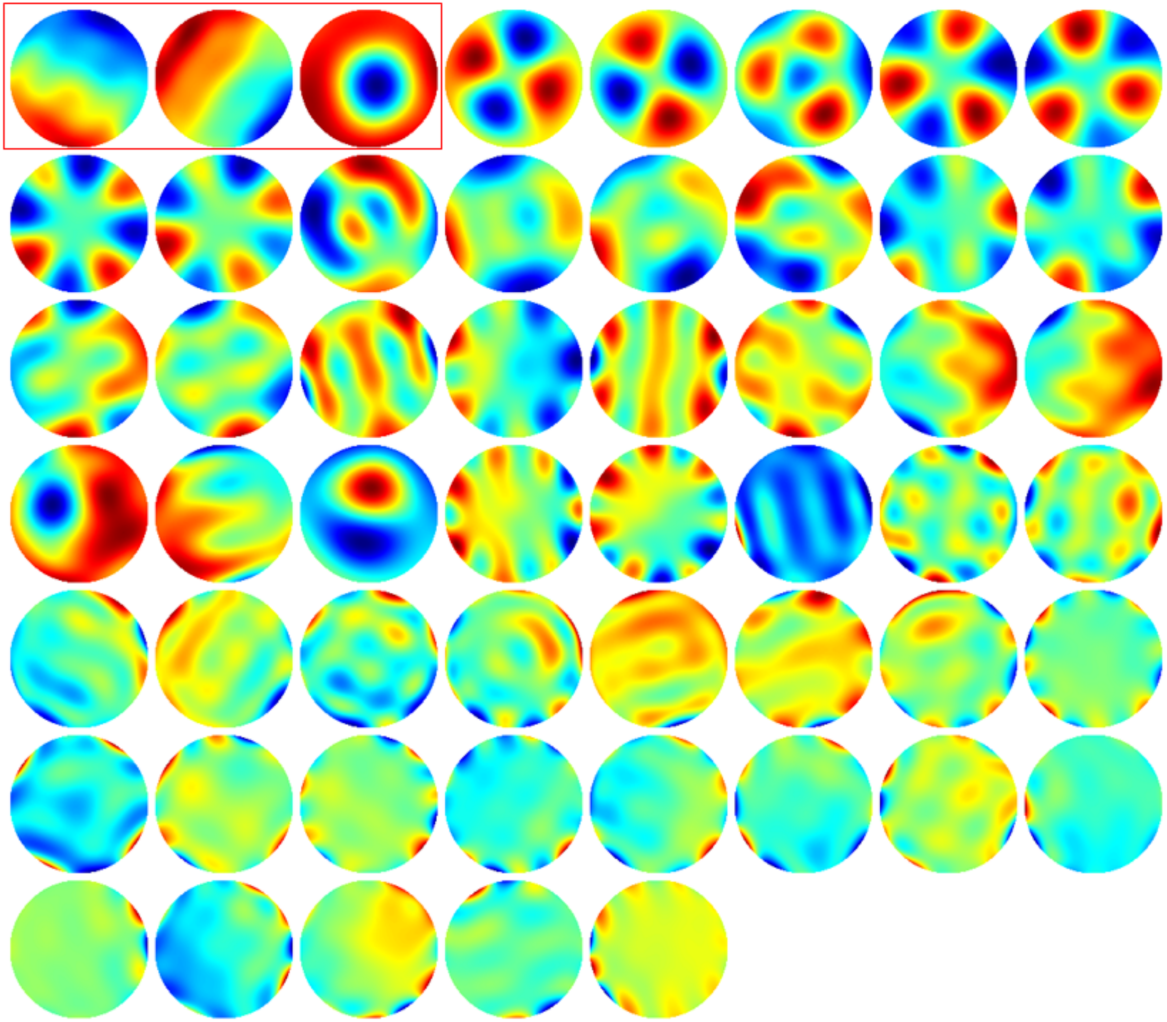
Experimental measurements of the normalized gradient-orthogonal base generated by a 69 actuators DM. In the red highlight, the three modes used for displacement, and therefore excluded from the aberration correction procedure.

Performing singular values decomposition on the matrix *G*, so that *G* = *USV*, the lines of matrix *V* are a set of coefficients, with singular values equal to the values of the diagonal of *S*, constituting a gradient orthogonal base for the AOE. For practical implementation of the correction method, the three elements of the base most similar to tip, tilt and defocus Zernike terms should be neglected, as well as elements of the base with neglectable singular values. The phase distribution of a representative gradient orthogonal base are reported in Fig 3.

It is to be noted that, depending on the design of the optical system, the image of the AOE on the back aperture of the microscopy system could be slightly larger than the optical aperture. If that is the case, this should be carefully taken into account, and computation of the gradient dot product as in Eq (28) should be only performed on the centroids within the aperture area effectively used in the microscope.

## Experimental results

In order to prove the proposed method works in experimental conditions, test measurements were performed both on a standard epifluorescence setup, and a multiaperture confocal microscope based on a digital micromirror device. Performances of the gradient orthogonal base were compared to the use of a simulated Zernike base, often used in hill-climbing optimization when no calibration is available [12, 13]. The epifluorescence microscope is a simple, low cost adaptive setup, based on LED excitation at 470*nm* (M470L3, Thorlabs, US), a 43 actuators piezoelectric DM (DMP40/M-P01, Thorlabs, US) and an industrial CMOS camera (UI-3060CP, IDS, Germany). An array of focal spots was generated in an image plane through a single mode solid state laser (sapphire 488 nm LP, Coherent, US), and a microlens array (MLA300-14AR-M). The spacing between illumiation spots was fixed by the geometry of the camera and the microlens array, resulting in a usable array of 38x24 spots, with a spacing of 300*μm* on the camera, resulting of a 7.5*μm* at the sample plane in experimental conditions. It is to be noticed that, while the laser source used has a maximum power of 100*mW*, power levels < 5*mW* were sufficient to perform the test measurements reported.

The pinhole based setup is a custom made adaptive multi aperture confocal microscope, recently used for a model free optimization application [14]. The setup is based on incoherent LED illumination (PT121B, Luminous, US), and a Digital Micromirror Device (Lightcrafter 6500 EVM, Texas Instruments, US) acting both as an array of point sources and as a confocal array of pinholes. Exploiting the binary programmable nature of the array, isolated active pixels can act as pinholes, while the surrounding inactive pinholes can act as a tilted reflective surface. A secondary camera (Optimos, QImaging, Canada) is used to image such plane. A static array of equally spaced pinholes is created on the DMD. The number and spacing of spots is customizable through the DMD. In experimental conditions, an array of 15 by 8 spots with a spacing of 812*μm* is generated at the camera plane, resulting in a spacing of approximately 24*μm* at the sample plane in experimental conditions. The images of all pinholes are cropped from the camera image, and averaged, and the second moment of the average distribution was computed according to Eq (6). The AOE used for aberration correction is a 69 magnetic actuators DM (DM-69, Alpao, France). Calibration of the DMs was performed with two separate Shack Hartmann detectors. The lower order piezoelectric DM of the epifluorescence microscope was calibrated with a CCD based wavefront sensor (WFS150-7AR, Thorlabs, US) with a resolution of 1280 × 1024 pixels, 5.95 × 4.76*mm*^2^ area sensor, with 150 *μm* pitch and 5*mm* focal length, for a total of approximately 700 subapertures in the pupil plane, while the higher order magnetic actuators DM for the confocal setup was calibrated with a high resolution Shack-Hartmann wavefront sensor (Flexible Optical B.V., the Netherlands) with a resolution of 2000 × 2000 pixels, 10 × 10 mm^2^ area sensor, with 63 *μm* pitch and 2*mm* focal length, for a total of approximately 18000 subapertures in the pupil plane. All measurements, on both microscopes were performed with a 40X, 1.25 numerical aperture, oil immersion objective (Leica, Germany), and a standard set of Green fluorescence protein filters (MDF-GFP, Thorlabs, U.S.). The sample used for experimental tests on the epifluorescence microscope is a prepared slide with BPAE cells, with microtubules stained with Bodipy (Fluocells prepared slide # 2, Invitrogen, US). The sample for experimental tests on the confocal microscope is a 16 *μm* thick prepared slide of mouse kidney stained with Alexa 488 (Fluocells prepared slide # 3, Invitrogen, US). Severe aberrations, for testing purposes, were introduced by drying a 2% agarose solution on the coverglass.

The calibration procedure of the DM resulted in the generation of a base of 50 gradient-orthogonal aberrations for the high order DM, and 32 for the low order DM, excluding tip, tilt and defocus. In order to verify that the base respects condition (4), with the high order DM, for every couple of base elements *φ*_*i*_, *φ*_*j*_, measurements of the metric were performed for aberrations *φ*_*ij*_ = *a*_*i*_*φ*_*i*_ + *a*_*j*_*φ*_*j*_, with −3*μm* < *a*_*i*_, *a*_*j*_ < 3*μm*, for 7 uniformly distributed values of *a*_*i*_ and *a*_*j*_, resulting in a total of 49 measurements per couple of base elements. The resulting functions were fitted with the quadratic model

$$\begin{array}{c} M\left(a_{i},a_{j}\right)=A_{1}{\left(a_{i}\cos\left(\theta \right)-a_{j}\sin\left(\theta \right)\right)}^{2}+A_{2}{\left(a_{j}\cos\left(\theta \right)+a_{i}\sin\left(\theta \right)\right)}^{2}+M_{0}. \end{array}$$ (30),

equivalent to a two dimensional version of Eq (3), which respects condition (4) in case the angle *θ* is Zero, or *A*_1_ = *A*_2_.

**Fig 4:**
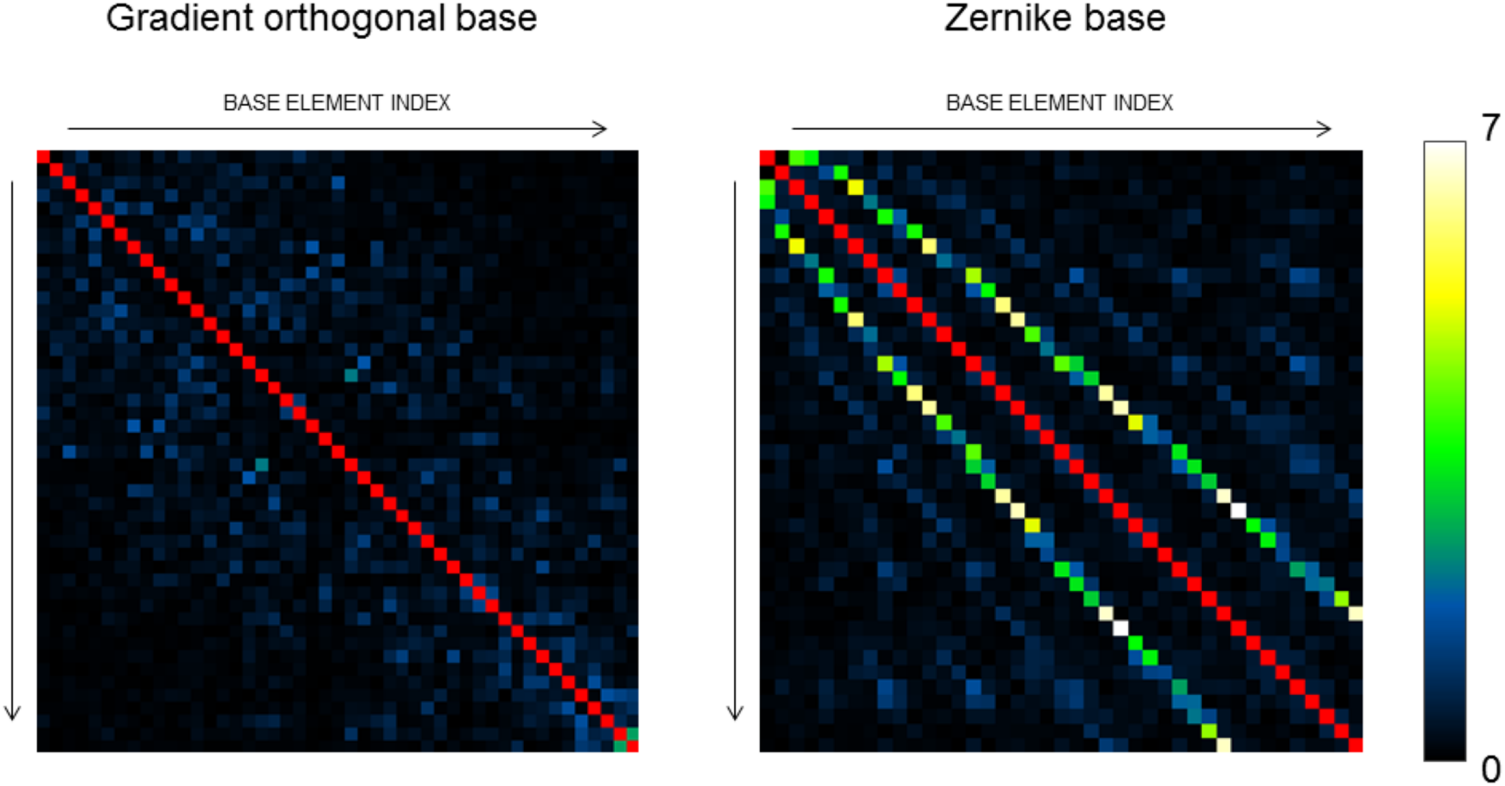
Experimental measurements of parameter *P* for an optimal, gradient orthogonal base and a Zernike base. The diagonal values are infinite, and therefore omitted.

In order to quantify the deviation of the metric function from (4), the parameter *P* was used:

$$\begin{array}{c} P\left(a_{i},a_{j}\right)=(\frac{\max(|A_{1}|,|A_{2}|)}{\min(|A_{1}|,|A_{2}|)}-1)\sin\left(2\theta \right), \end{array}$$ (31)

which is equal to zero if *φ*_*i*_ and *φ*_*j*_ respect condition (4), and infinite if *φ*_*i*_ ∝ *φ*_*j*_. As a term of comparison, the same measurement was performed on a simulated Zernike base up to the eighth order. Results are reported in Fig 4.

It can be observed that for Zernike polynomials every base element has at least one clearly not orthogonal other base member. For the optimal base, as expected, the values of parameter *P* have much lower values, and are generally randomly distributed.

**Fig 5:**
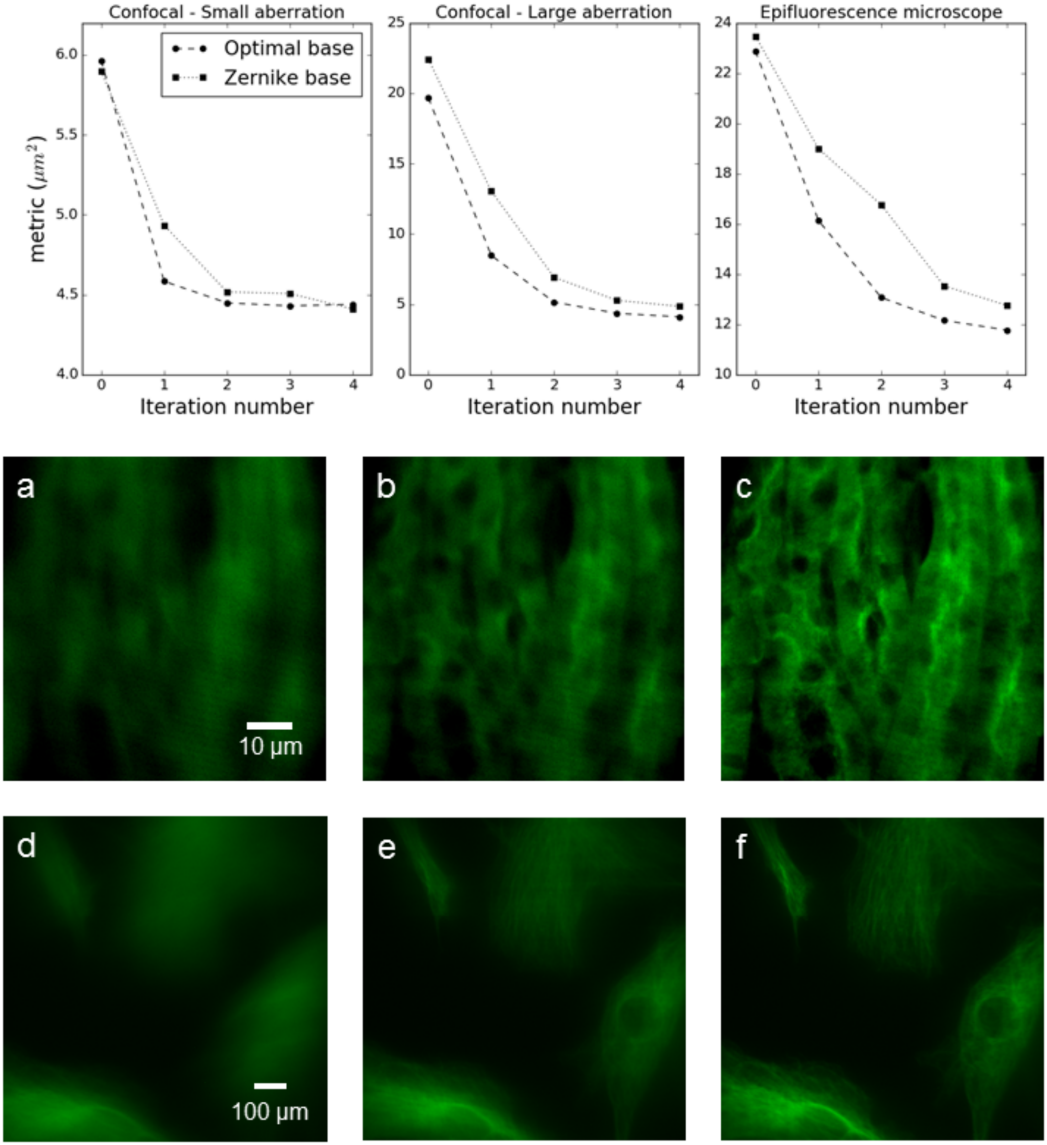
Representative results of optimization for small and severe aberrations. Images reported are: a- Confocal image for non compensated severe aberration. b- Confocal image for severe aberration after two correction iterations with Zernike base. c- Confocal image for severe aberration after two correction iterations with gradient orthogonal base. d- Epifluorescence image for non compensated severe aberration. e- Epifluorescence image for severe aberration after two correction iterations with Zernike base. f- Epifluorescence image for severe aberration after two correction iterations with gradient orthogonal base.

In order to verify the performance of aberration correction, sequential 2*N* + 1 measurements corrections as described in the introduction were performed on the samples with severe aberrations introduced by an agarose layer. In both microscopes, optimization was performed at 100*Hz*, resulting in an optimization time of 0.65*s* for the low order epifluorescence microscope, and 1.01*s* for the high order confocal microscope. In the case of the confocal microscope, the system was tested separately with a clear coverslip, correcting for the sample induced aberration at a depth of ≈ 10*μm*. The same test could not be performed on the epifluorescence microscope, as the sample did not show any detectable sample induced aberration. Usage of the gradient orthogonal base proved to produce the optimal correction in a single iteration for small aberrations, and in two iterations for more severe aberrations, possibly due to the experimental error in the metric calculation and in the fit of the metric function. On the other hand, performing the same correction procedure with a Zernike base, even small aberrations required several iterations to reach optimal correction. Some representative results are reported in Fig 5.

## Conclusion

In this paper, a novel sensorless method for modal aberration correction in fluorescence microscopes has been presented. The method is based on sample-independent pre-calibration of an orthogonal set of modes, describing the connection between the adaptive optics action and the performance metric expressed as the second moment of the fluorescence distribution detected in epifluorescence from a point-like excitation pattern. As a result of such pre-calibration, the optimal correction can be achieved in 2*N* + 1 measurements of the metric, for aberrations of any amplitude. The methodology can be implemented, with the addition of a secondary excitation source producing an array of point-like spots, in camera based systems (e.g. epifluorescence microscope, structured illumination microscope, localization based superresolution) or, with the addition of a reflective aperture surface and a camera detector, in aperture-based confocal systems (e.g. laser scanning confocal, spinning disk microscopes, STED). The validity of the method has been experimentally proven in epifluorescence microscopy, and on a programmable multiaperture confocal microscope.
